# Generation of a bovine cell line for gene engineering using an HIV-1-based lentiviral vector

**DOI:** 10.1038/s41598-022-20970-6

**Published:** 2022-10-18

**Authors:** Nanami Morizako, Erika P. Butlertanaka, Yuri L. Tanaka, Honoka Shibata, Tamaki Okabayashi, Hirohisa Mekata, Akatsuki Saito

**Affiliations:** 1 Department of Veterinary Science, Faculty of Agriculture, University of Miyazaki, Miyazaki, Miyazaki 8892192 Japan; 2 Center for Animal Disease Control, University of Miyazaki, Miyazaki, Miyazaki 8892192 Japan; 3 Graduate School of Medicine and Veterinary Medicine, University of Miyazaki, Miyazaki, Miyazaki 8891692 Japan

**Keywords:** Biotechnology, Microbiology

## Abstract

Human immunodeficiency virus type 1 (HIV-1)-based lentiviral vectors are indispensable tools for gene engineering in mammalian cells. Conversely, lentiviral vector transduction is severely inhibited in bovine cells. Previous studies demonstrated that this inhibition is caused by the anti-lentiviral host factor tripartite motif containing 5 (TRIM5), which targets incoming HIV-1 virions by interacting with the viral capsid. In this study, we investigated several methods for overcoming the limited applicability of lentiviral vectors in bovine cells. First, we demonstrated that the SPRY domain of bovine TRIM5 is the major determinant of anti-viral activity. Second, we found that mutations that allow the capsid to evade rhesus macaque TRIM5α minimally rescued HIV-1 infectivity in bovine-derived MDBK cells. Third, we found that cyclosporine A, which relieves the inhibition of HIV-1 infection in monkey cells, significantly rescued the impaired HIV-1 infectivity in MDBK cells. Lastly, we successfully generated a bovine cell line lacking intact *TRIM5* using the CRISPR/Cas9 technique. This *TRIM5* knockout cell line displayed significantly higher susceptibility to an HIV-1-based lentiviral vector. In conclusion, our findings provide a promising gene engineering strategy for bovine cells, thereby contributing to innovations in agriculture and improvements in animal health.

## Introduction

Although mouse leukemia virus (MLV)-based retroviral vectors are powerful tools for gene engineering in mammalian cells, the vectors require cellular mitosis for efficient infection^1,2^. Therefore, it is technically challenging to use retroviral vectors for non-dividing cells such as neural cells, macrophages, and dendritic cells. By contrast, human immunodeficiency virus type 1 (HIV-1)-based lentiviral vectors efficiently infect both dividing and non-dividing cells^1,3^. A previous study demonstrated that cone-shaped HIV-1 capsid (CA) proteins translocate through nuclear pore complexes^4^. Thus, HIV-1-based lentiviral vector has been widely used in broad fields including gene therapy in humans and gene modification of animals^5^. However, transduction by HIV-1-based lentiviral vectors is severely inhibited in bovine cells during the step of reverse transcription^6,7^, hampering this strategy. This inhibition is caused by an anti-HIV-1 host factor tripartite motif containing 5 (TRIM5) in bovine cells^6,7^, which targets incoming HIV-1 virions by interacting with viral CA. TRIM5α was initially identified as an anti-HIV-1 host factor in rhesus macaque (RM) cells^8^. Mammalian TRIM5 consists of a RING domain, B-box domain, Coiled-coil domain, and SPRY (B30.2) domain^9^. The RING domain is a class E3 ubiquitin ligase that is involved in the proteasome-mediated viral CA degradation^10^. The B-box and Coiled-coil domains are responsible for the dimerization and higher-order association of TRIM5 protein^11–13^. The SPRY domain is involved in direct interaction with viral CA^14^. Importantly, *TRIM5* gene evolved under positive selection^15^, suggesting that mammalian *TRIM5* has been the frontline anti-retroviral host factor.

In this study, we investigated multiple methods for overcoming the limited applicability of HIV-1-based lentiviral vectors in bovine cells. First, we demonstrated that the SPRY domain of bovine TRIM5 was the major determinant of anti-viral activity. Second, we found that CA mutations that confer resistance to RM TRIM5α or owl monkey TRIMCyp failed to rescue HIV-1 infectivity in bovine-derived Madin–Darby bovine kidney (MDBK) cells. Third, we demonstrated that cyclosporine A (CsA), which relieves the inhibition of HIV-1 infection in monkey cells^16,17^, could rescue the impaired HIV-1 infectivity in MDBK cells. Finally, we generated a MDBK cell line lacking intact *TRIM5* using the CRISPR/Cas9 method. *TRIM5*-knockout cells exhibited significantly greater susceptibility to HIV-1 infection. Furthermore, we demonstrated that an HIV-1 vector efficiently infected cell cycle-arrested *TRIM5*-knockout cells. Overall, our methods developed in this study provide a promising strategy for gene engineering in bovine cells, contributing to innovations in agriculture and improvements in animal health.

## Results

### *TRIM5* depletion sensitized bovine-derived MDBK cells to HIV-1 infection

**Figure 1:**
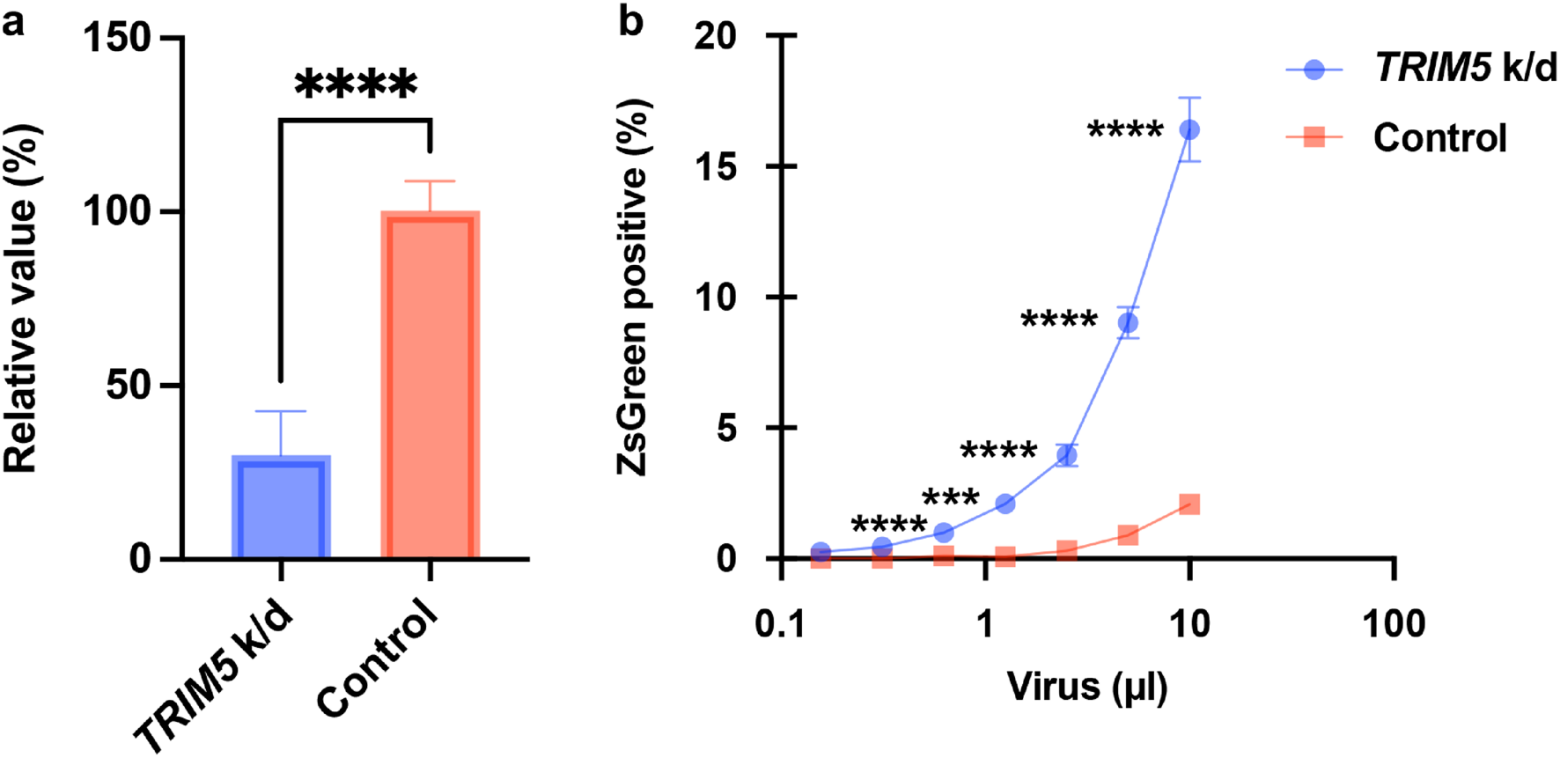
Significant effects of bovine TRIM5 on the inhibition of HIV-1 vectors in MDBK cells. (**a**) The mRNA expression of *TRIM5* in MDBK cells transfected with siRNA targeting *TRIM5* or control siRNA was measured by qRT-PCR. (**b**) MDBK cells transfected with siRNA targeting *TRIM5* or control siRNA were infected with different amounts of HIV-1 vectors encoding ZsGreen reporter protein. The fluorescence, which is a surrogate marker of infection, was measured using a flow cytometer 2 days after infection. The results are presented as the mean and standard deviation of triplicate measurements from one assay, and they are representative of at least three independent experiments. Differences were examined by a two-tailed, unpaired Student’s *t*-test. ****p < 0.0001, ***p < 0.001.

To investigate the impact of bovine TRIM5 on inhibition of HIV-1 infection in bovine cells, we depleted *TRIM5* in MDBK cells via siRNA transfection. We used siRNA targeting bovine *TRIM5* as previously described^6^. Prior to testing the anti-viral activity, the efficiency of *TRIM5* depletion was evaluated by quantitative real-time reverse transcription (qRT)-PCR. The results in Fig. 1a illustrate that siRNA transfection decreased *TRIM5* mRNA levels by 75% versus those in normal MDBK cells. To test the effect of *TRIM5* depletion, the cells were infected with an HIV-1-based vector encoding ZsGreen reporter protein. Our flow cytometric analysis revealed that the infectivity in *TRIM5*-depleted MDBK cells was approximately tenfold higher than that in mock-transfected MDBK cells, demonstrating that bovine TRIM5 was a major anti-HIV-1 host factor in MDBK cells.

### The SPRY domain is the determinant for the anti-viral activity of bovine TRIM5

**Figure 2:**
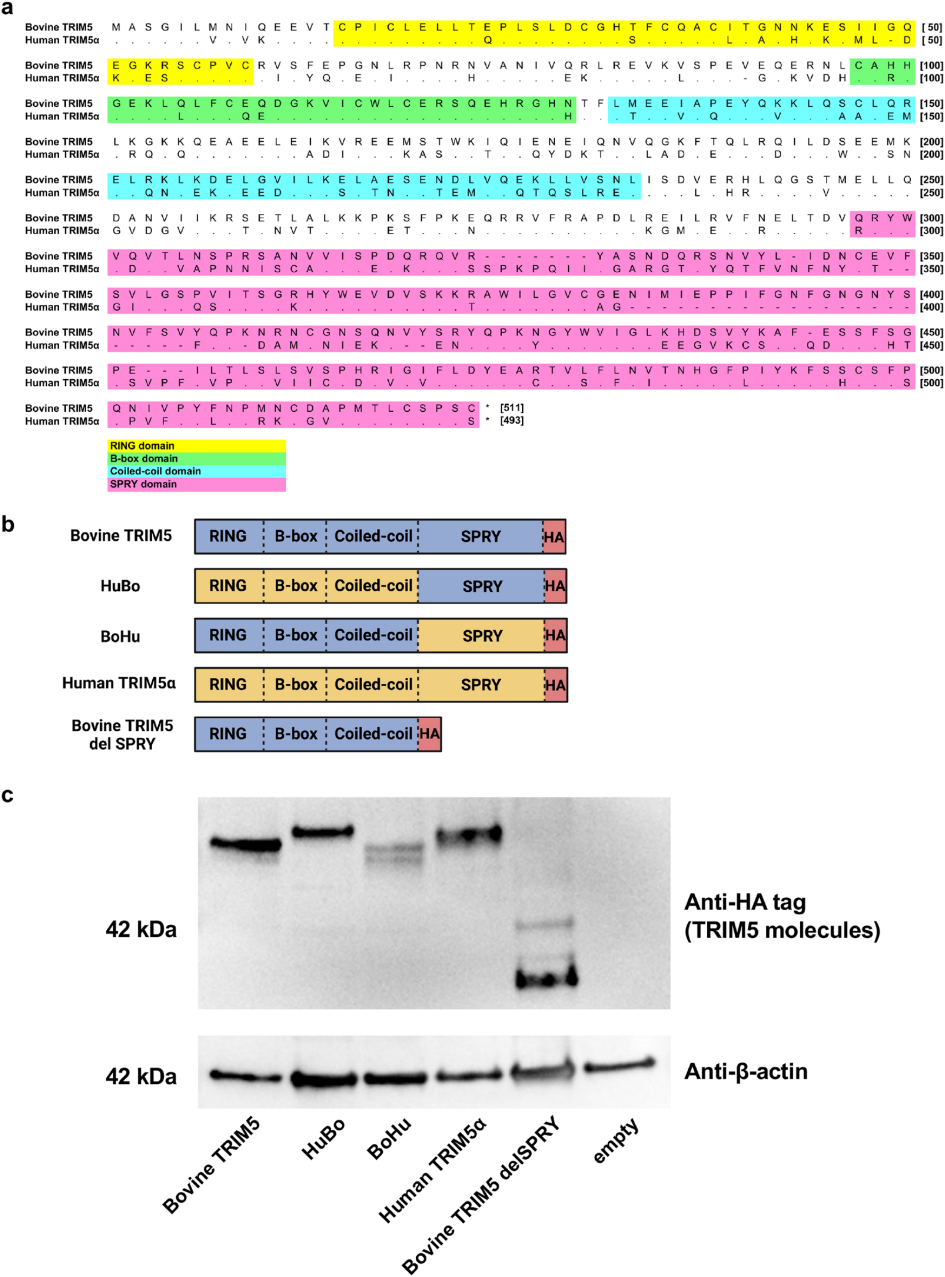
Generation of CRFK cells expressing chimeric TRIM5 proteins. (**a**) Alignment of bovine TRIM5 and human TRIM5α. The protein sequences of bovine TRIM5 and human TRIM5α were aligned using MEGA X. The domains of TRIM5 were indicated in yellow (RING domain), green (B-box domain), blue (Coiled-coil domain), and pink (SPRY domain). (**b**) Schematic representation of chimeric TRIM5 molecules generated in this study. The chimera TRIM5 molecule “HuBo” consists of the RING, B-box, and Coiled-coil (RBCC) domains of human TRIM5α and the SPRY domain of bovine TRIM5. The chimeric TRIM5 molecule “BoHu” consists of the RBCC domain of bovine TRIM5 and SPRY domain of human TRIM5α. A truncated version of bovine TRIM5 lacking the SPRY domain was termed “delSPRY.” (**c**) Expression of each HA-tagged TRIM5 protein in CRFK cells was determined using Western blotting. The cellular lysate of unmodified CRFK cells was used as a negative control (empty).

**Figure 3:**
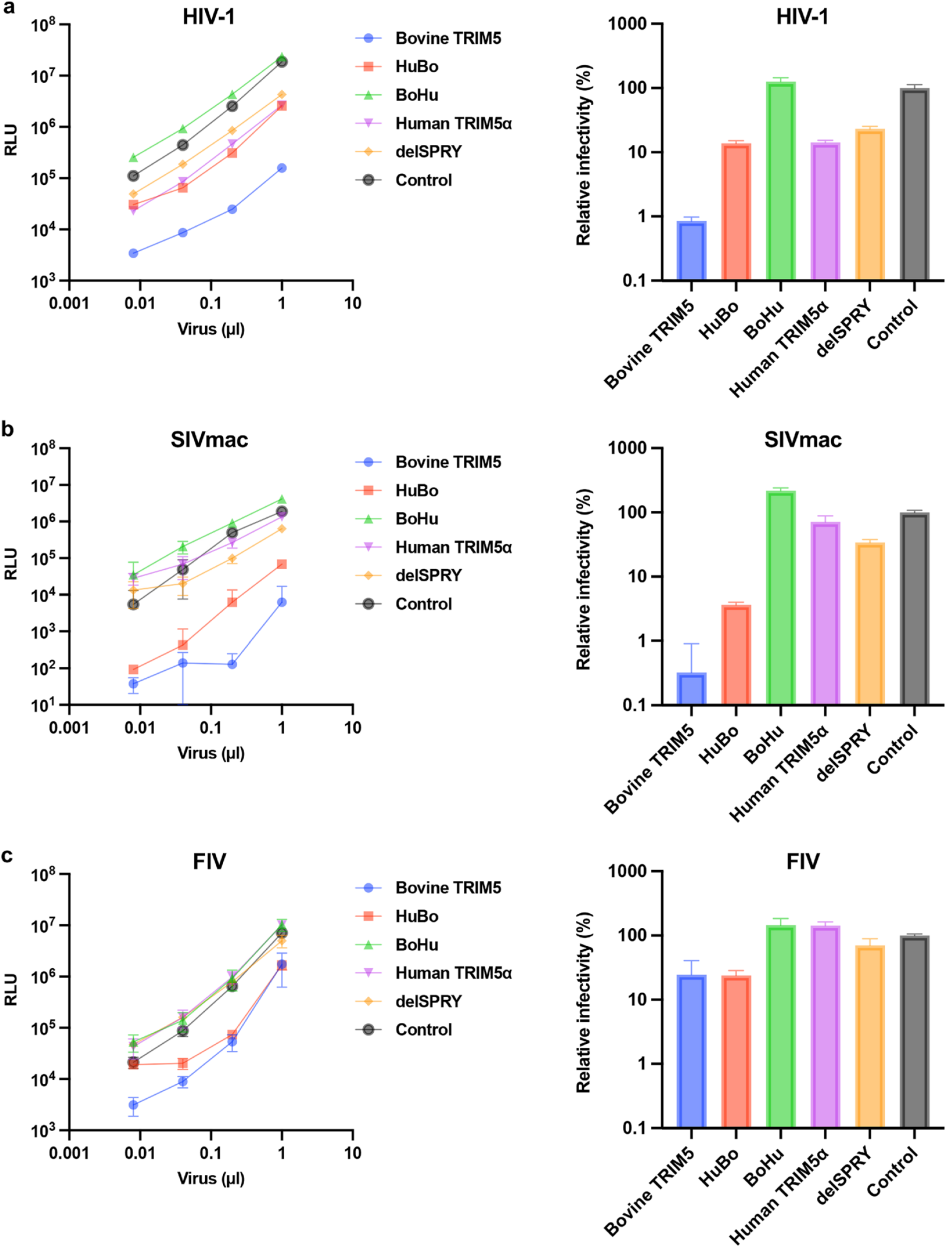

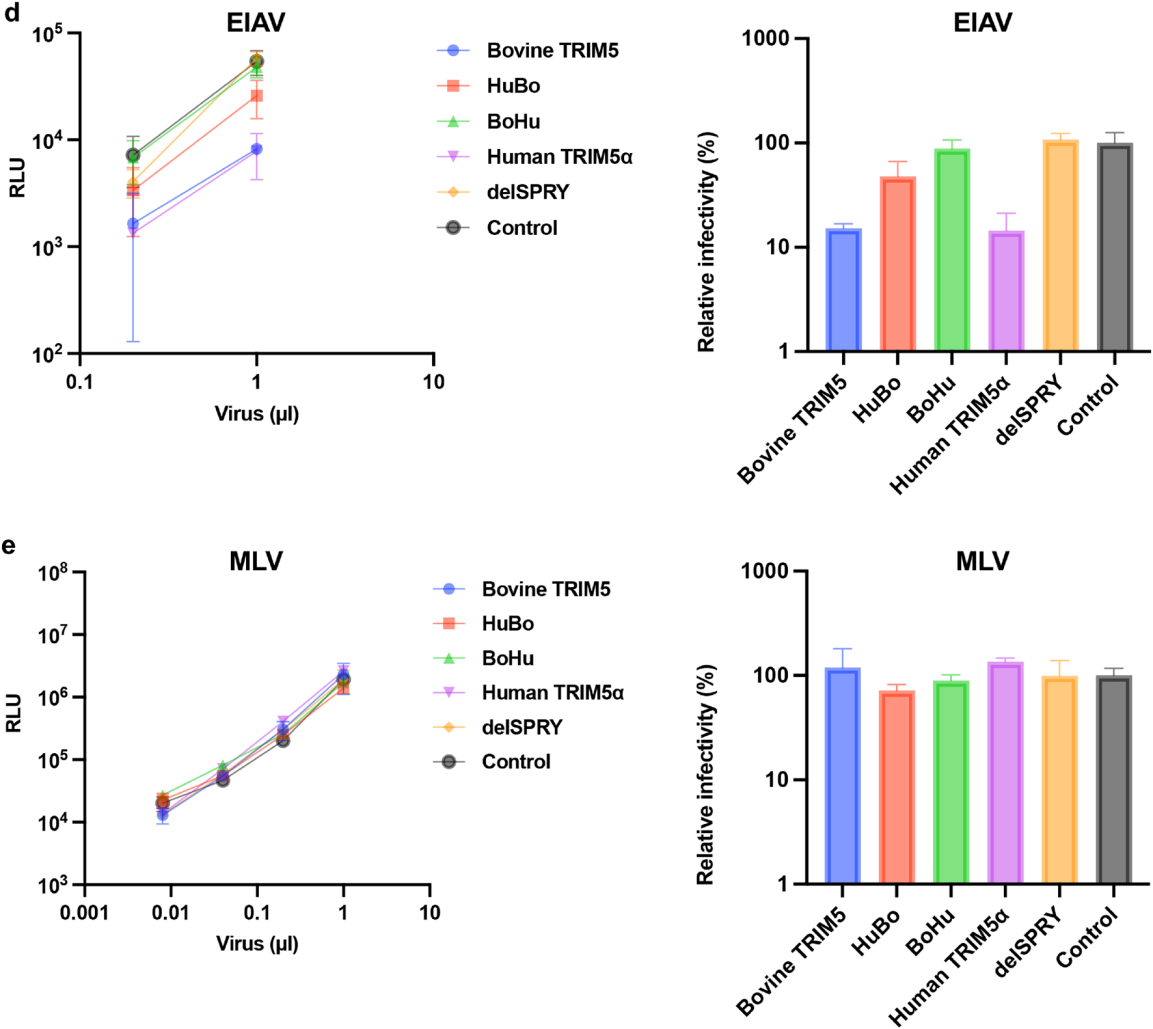
The SPRY domain of bovine TRIM5 is the determinant of anti-lentiviral activity. (**a**) (left) CRFK cells expressing TRIM5 proteins were infected with an HIV-1 vector encoding luciferase reporter protein. The infectivity was determined as relative light units (RLU) 2 days after infection. (right) Relative infectivity was calculated using the values in the left panel. (**b**) (left) CRFK cells expressing TRIM5 proteins were infected with an SIVmac vector encoding luciferase reporter protein. The infectivity was determined as relative light units (RLU) 2 days after infection. (right) Relative infectivity was calculated using the values in the left panel. (**c**) (left) CRFK cells expressing TRIM5 proteins were infected with an FIV vector encoding luciferase reporter protein. The infectivity was determined as relative light units (RLU) 2 days after infection. (right). Relative infectivity was calculated using the values in the left panel. (**d**) (left) CRFK cells expressing TRIM5 proteins were infected with an EIAV vector encoding luciferase reporter protein. The infectivity was determined as relative light units (RLU) 2 days after infection. (right) Relative infectivity was calculated using the values in the left panel. (**e**) (left) CRFK cells expressing TRIM5 proteins were infected with an MLV vector encoding luciferase reporter protein. The infectivity was determined as relative light units (RLU) 2 days after infection. (right) Relative infectivity was calculated using the values in the left panel.

We demonstrated that bovine TRIM5 strongly inhibited HIV-1 infection (Fig. 1b). By contrast, previous studies demonstrated that HIV-1 evades restriction by human TRIM5α^8^. The identity between bovine TRIM5 and human TRIM5α was 56.0% (calculated on the website of SIM—Alignment Tool for protein sequences [https://web.expasy.org/sim/], Fig. 2a). To identify the TRIM5 domain that determines the species specificity, we generated a series of chimeric TRIM5 molecules between bovine TRIM5 and human TRIM5α (Fig. 2b). Whereas the molecule containing the human TRIM5α-derived RING, B-box, and Coiled-coil domains (RBCC) and bovine-derived SPRY domain was termed “HuBo,” chimeric TRIM5 containing a bovine TRIM5-derived RBCC domain and a human TRIM5α-derived SPRY domain was termed “BoHu.” Furthermore, we generated a truncated form of bovine TRIM5 lacking the SPRY domain (termed “delSPRY”). We used a *TRIM5*-null Crandell–Rees feline kidney (CRFK) cell line to generate cells stably expressing these molecules. After confirming the expression of each TRIM5 molecule by Western blotting (Fig. 2c and Supplemental Fig. 1), the cells were infected with HIV-1 vectors encoding luciferase reporter protein. Consistent with the observation in MDBK cells (Fig. 1b), HIV-1 infection was significantly blocked in CRFK cells expressing bovine TRIM5 (blue) (Fig. 3a). Conversely, human TRIM5α (purple) slightly blocked HIV-1 infection, reproducing the previous findings^8,18–21^. In CRFK cells expressing chimeric molecules, although viral infection was significantly suppressed in CRFK cells expressing HuBo (red), infection was not blocked in CRFK cells expressing BoHu (green). Furthermore, CRFK cells expressing delSPRY (orange) displayed significantly weaker inhibition than those expressing bovine TRIM5 (blue). These observations suggest that the SPRY domain of bovine TRIM5 is the major determinant of the TRIM5-mediated inhibition of HIV-1 infection.

In addition to HIV-1 vectors, the anti-viral activity of chimeric TRIM5 molecules against other lentiviruses including simian immunodeficiency virus (SIVmac, Fig. 3b), feline immunodeficiency virus (FIV, Fig. 3c), and equine infectious anemia virus (EIAV, Fig. 3d) was examined. Whereas TRIM5 molecules containing bovine SPRY (blue and red) suppressed infection by SIVmac and FIV (Fig. 3b–c), those carrying human SPRY domain (green and purple) minimally blocked these viruses. Consistent with previous reports^6,7,22^, both bovine TRIM5 and human TRIM5α blocked EIAV infection (Fig. 3d). Interestingly, both chimeric molecules (red and green) lost anti-EIAV activity. To test the specificity of inhibition by these TRIM5 molecules, the infectivity of MLV was examined in these cells. We observed comparable infectivity of MLV on all cells (Fig. 3e), supporting that the obtained results indicated specific inhibition by TRIM5 molecules. Collectively, bovine TRIM5 exhibits broad anti-lentiviral activity, and the SPRY domain is the major determinant of its anti-viral activity.

### Mutations in CA did not rescue HIV-1 infection in MDBK cells

**Figure 4:**
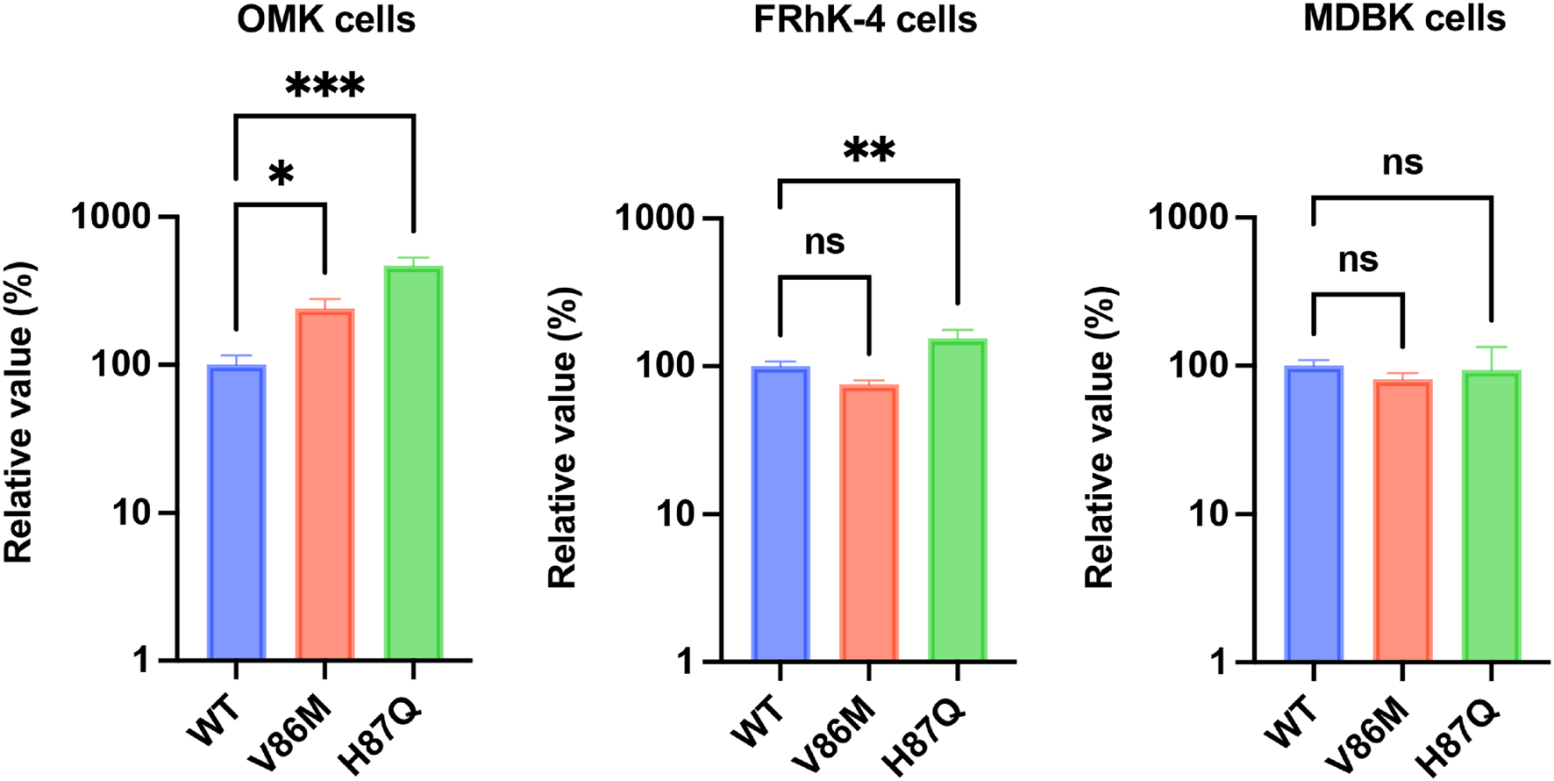
HIV-1 CA mutants with resistance to RM TRIM5 failed to evade inhibition in MDBK cells. OMK (left), FRhK-4 cells (center), and MDBK cells (right) were infected with WT, V86M, or H87Q CA mutants encoding luciferase reporter protein. The infectivity was determined as relative light units (RLU) 2 days after infection. Relative infectivity was calculated using the values of the WT virus. The results are presented as the mean and standard deviation of triplicate measurements from one assay, and they are representative of at least three independent experiments. Differences were examined by one-way ANOVA, followed by the Tukey test. ***p < 0.001, **p < 0.01, *p < 0.05, ns (not significant).

Previous research demonstrated that viral CA of incoming HIV-1 particles is the interface recognized by mammalian TRIM5 molecules [reviewed in ^23^]. Therefore, mutations in CA can alter the susceptibility to TRIM5-mediated inhibition. Previous studies demonstrated that long-term passaging of wild-type (WT) HIV-1 in a cell line expressing RM TRIM5α resulted in the selection of the V86M or H87Q mutation in the cyclophilin A (CypA)-binding loop of CA^24,25^. Importantly, these mutations partially rescued restriction by RM TRIM5α^25,26^. To investigate whether V86M and H87Q CA mutants could evade inhibition by bovine TRIM5, we used owl monkey kidney (OMK) cells, RM-derived FRhK-4 cells, and MDBK cells for infection experiments because HIV-1 infection was blocked in these cell lines. The result demonstrated that both V86M and H87Q exhibited higher infectivity than WT HIV-1 in OMK cells (Fig. 4). In FRhK-4 cells, the H87Q mutation partially rescued HIV-1 infection. However, neither V86M nor H87Q rescued HIV-1 infection in MDBK cells. Collectively, CA mutations that confer partial resistance to HIV-1 in monkey cells failed to rescue HIV-1 infectivity in MDBK cells.

### CsA treatment significantly rescued HIV-1 infectivity in MDBK cells

**Figure 5:**
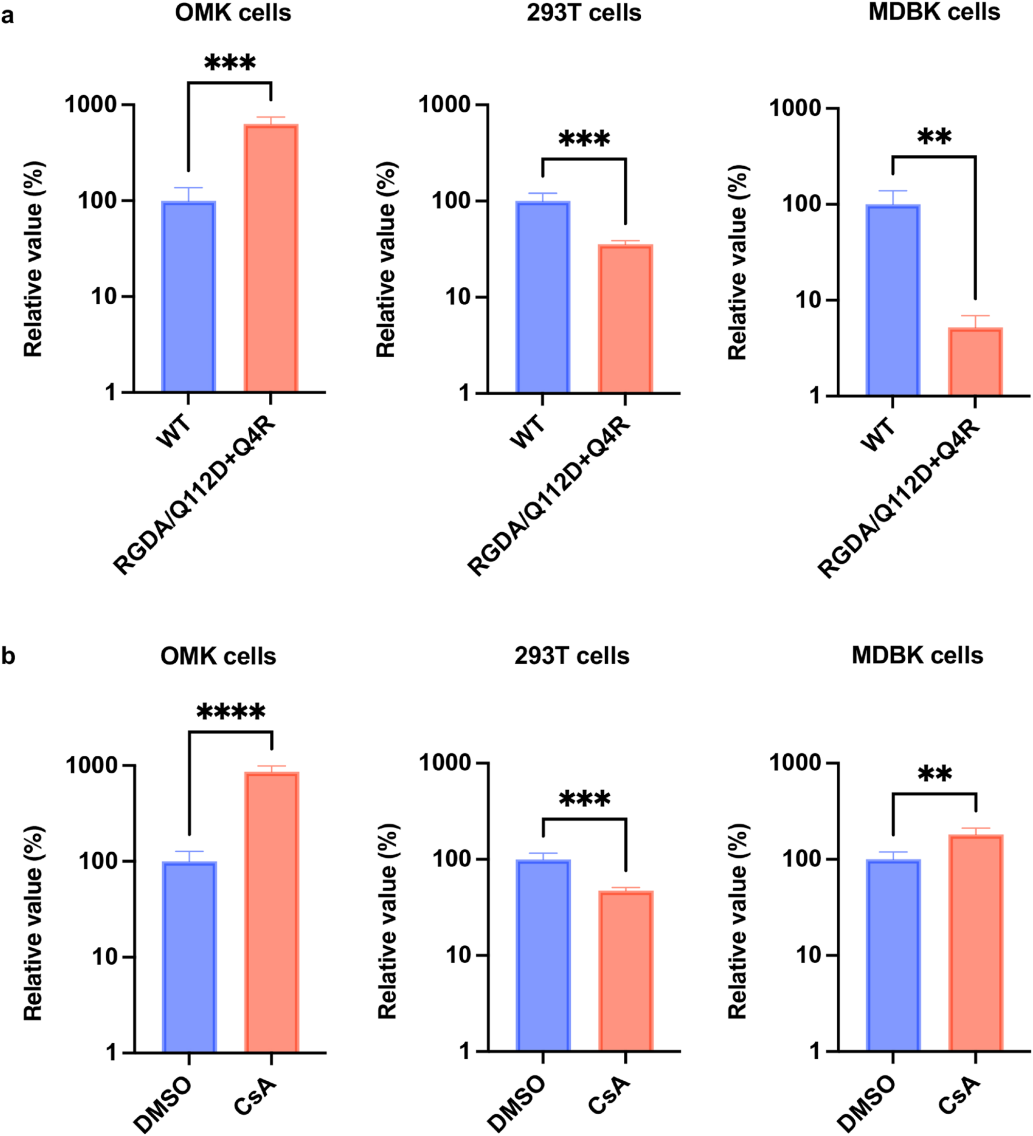
CsA treatment significantly enhanced HIV-1 infectivity in MDBK cells. (**a**) OMK (left), Lenti-X 293T (center), and MDBK cells (right) were infected with the WT virus or RGDA/Q112D + Q4R CA mutant encoding luciferase reporter protein. The infectivity was determined as relative light units (RLU) 2 days after infection. Relative infectivity was calculated using the values of the WT virus. (**b**) OMK (left), Lenti-X 293T (center), and MDBK cells (right) were infected with HIV-1 vectors encoding luciferase reporter protein in the presence of DMSO or 2 µM Cyclosporin A (CsA). The infectivity was determined as RLU 2 days after infection. The relative infectivity was calculated according to the control values. The results are presented as the mean and standard deviation of triplicate measurements from one assay, and they are representative of at least three independent experiments. Differences were examined by a two-tailed, unpaired Student’s *t*-test. ****p < 0.0001, ***p < 0.001, **p < 0.01.

Previous studies demonstrated that cellular Cyclophilin A (CypA) is associated with TRIM5α-mediated inhibition^16,17,27^. In human CD4 + T cells, CypA protects HIV-1 from human TRIM5α^28^. Conversely, CypA sensitizes HIV-1 to TRIM5α in monkey cells^17^; thus, interruption of the CA–CypA interaction rescued HIV-1 infectivity in monkey cells. Based on these observations, we tested whether the disruption of CypA–CA binding rescues HIV-1 infection in bovine cells. To this end, we used two methods. We used a CA mutant, RGDA/Q112D + Q4R, possessing six mutations (Q4R, H87R, A88G, P90D, P93A, and Q112D) in CA^29^. We previously demonstrated that the RGDA/Q112D + Q4R mutant completely lost CypA binding. Furthermore, this CA mutant had higher infectivity than the WT virus in human-derived Jurkat cells. OMK, Lenti-X 293T, and MDBK cells were infected with WT or RGDA/Q112D + Q4R virus. The RGDA/Q112D + Q4R virus had an eightfold higher infectivity than the WT virus in OMK cells (Fig. 5a). This result can be explained by the fact that OMK cells express a TRIMCyp fusion protein between TRIM5α and CypA^16,30^. Conversely, the infectivity of RGDA/Q112D + Q4R was significantly lower than WT in Lenti-X 293T and MDBK cells. This suggests that although the RGDA/Q112D + Q4R mutations conferred resistance to TRIMCyp in OMK cells, these mutations sensitized HIV-1 to restriction factors in human and bovine cells.

It has been demonstrated that the inhibition of HIV-1 infection in OMK cells was canceled by treatment with Cyclosporin A (CsA), which specifically disrupts the CypA–CA interaction^16,30,31^. Based on this finding, we tested whether CsA treatment rescued HIV-1 infectivity in MDBK cells. OMK, Lenti-X 293T, and MDBK cells were infected with an HIV-1 vector in the presence or absence of CsA. The infectivity of the HIV-1 vector was increased by approximately 100-fold by CsA treatment in OMK cells (Fig. 5b), reproducing previous findings^16,30,31^. Contrarily, CsA treatment significantly decreased HIV-1 infectivity in Lenti-X 293T cells. Notably, HIV-1 infectivity was threefold higher in CsA-treated MDBK cells than in control cells. This suggests that host factors targeted by CsA, at least in part, are involved in the restriction of HIV-1 in MDBK cells. Collectively, CsA treatment significantly rescued HIV-1 infection in MDBK cells.

### Knockout of bovine *TRIM5* significantly enhanced HIV-1 infection on MDBK cells

**Figure 6:**
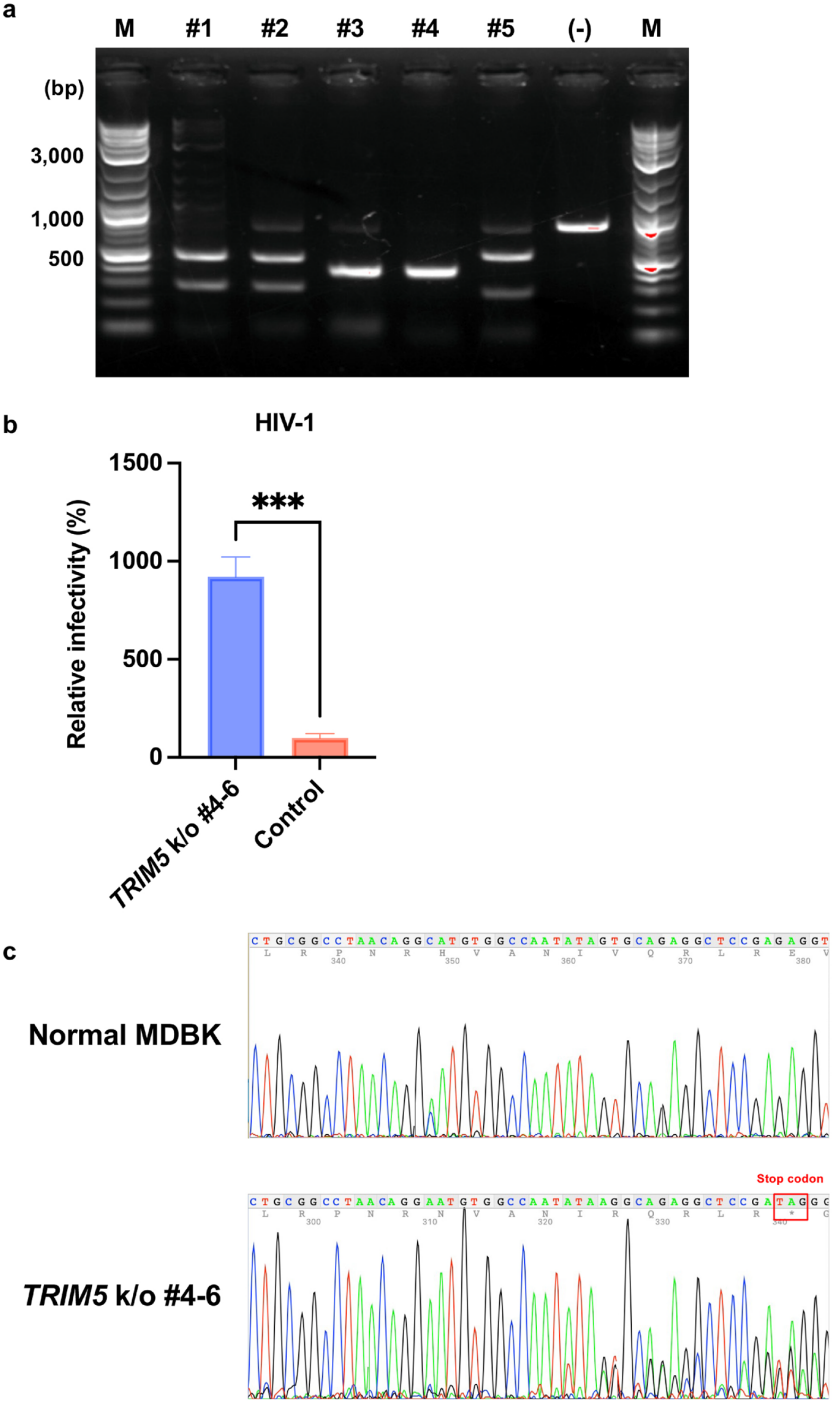
Knockout of *TRIM5* sensitized MDBK cells to HIV-1 infection. (**a**) Genomic DNA of MDBK cells encoding *TRIM5* was amplified using PCR. The amplicon was mixed with sgRNA and Cas9 protein to determine the cleavage efficiency. The results of electrophoresis are presented. “M” denotes marker, and “(-)” indicates an amplicon mixed with only Cas9 protein. (**b**) MDBK *TRIM5*-knockout (k/o) cells (clones #4–6) or normal MDBK cells (Control) were infected with HIV-1 vectors encoding luciferase reporter protein. The infectivity was determined as relative light units (RLU) 2 days after infection. Relative infectivity was calculated using the control values. The results are presented as the mean and standard deviation of triplicate measurements from one assay, and they are representative of at least three independent experiments. Differences were examined by a two-tailed, unpaired Student’s *t*-test. ***p < 0.001. (c) Genomic DNA of MDBK *TRIM5* k/o cells (clones #4–6) was amplified using PCR. The amplicon was subjected to sequencing to identify gene editing. The raw data on 4Peaks software (Nucleobytes) are presented.

Based on the aforementioned findings, we next knocked out *TRIM5* in MDBK cells. First, we screened five candidate single-guide RNA (sgRNA) constructs. We PCR-amplified a 796-base pair (bp) fragment covering the target sequences of the five sgRNAs. An in vitro cleavage assay demonstrated that sgRNA #4 cleaved the PCR fragment encoding bovine *TRIM5* with the highest efficiency (Fig. 6a). We therefore ligated this sgRNA sequence to the PX459 vector to generate PX459-TRIM5-sgRNA#4. Then, MDBK cells were transduced with PX459-TRIM5-sgRNA#4. After selection and single-cell cloning, each clone was evaluated by infection with HIV-1 vector encoding luciferase reporter protein. As presented in Fig. 6b, MDBK *TRIM5*-knockout cells (clones #4–6) exhibited approximately eightfold higher infectivity than the original MDBK cells. We next examined the genomic sequence of MDBK *TRIM5*-knockout cells (clones #4–6). The result demonstrated that this clone featured the deletion of one nucleotide, resulting in the emergence of an early stop codon (Fig. 6c).

**Figure 7:**
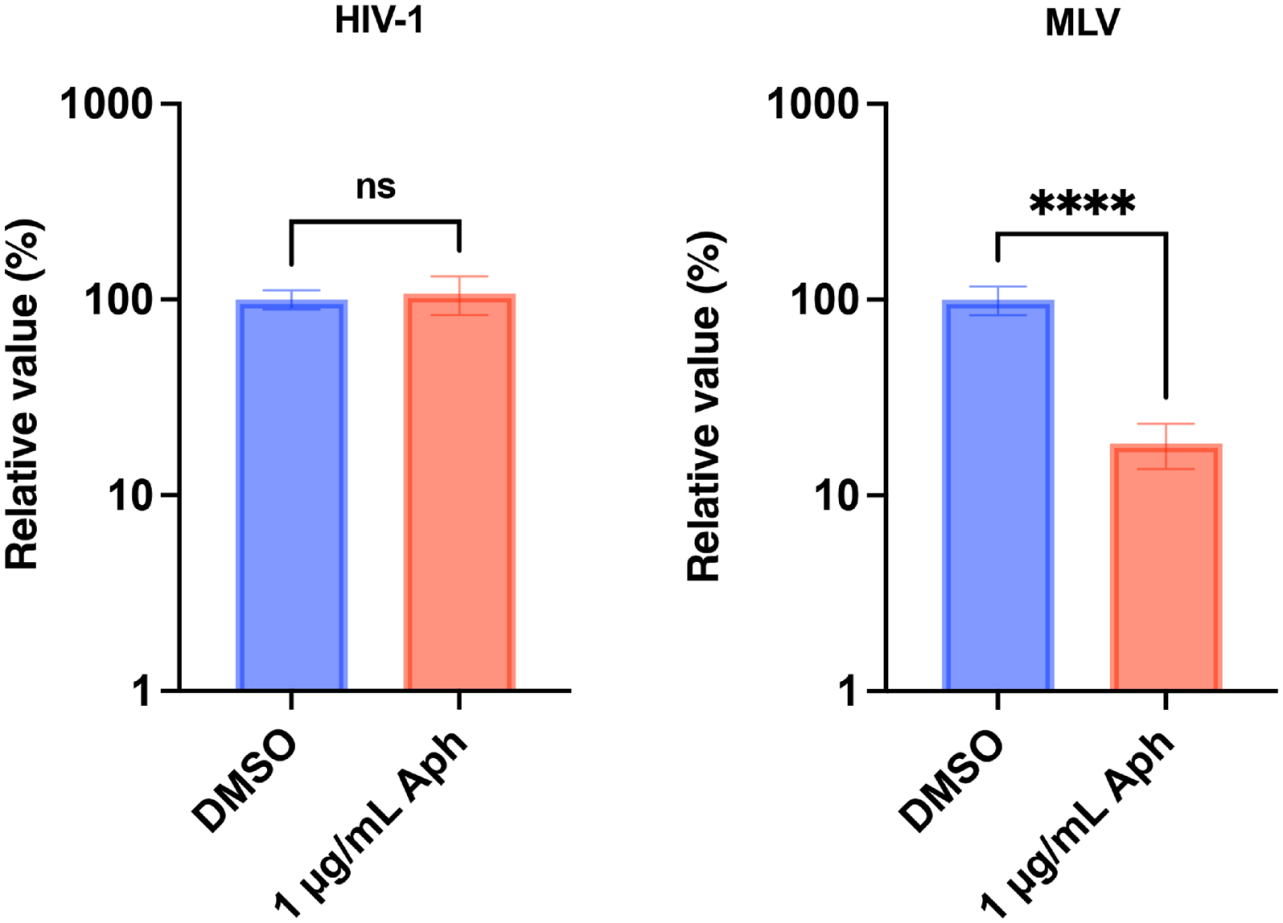
Efficient HIV-1 infection in non-dividing bovine cells. MDBK *TRIM5* k/o cells (clones #4–6) cultured in the presence of DMSO or 1 µg/mL Aphidicolin (Aph) were infected with an HIV-1 or MLV vector encoding ZsGreen reporter protein. The infectivity was measured using a flow cytometer 2 days after infection. The results are presented as the mean and standard deviation of hexaplicate measurements from one assay, and they are representative of at least three independent experiments. Differences were examined by a two-tailed, unpaired Student’s *t*-test. ****p < 0.0001, ns (not significant).

Next, we examined whether an HIV-1-based lentiviral vector could infect non-dividing cells. To this end, MDBK *TRIM5*-knockout cells (clones #4–6) were treated with aphidicolin (Aph) to arrest the cell cycle and infected with HIV-1 or MLV vectors. Consistent with previous observations^1,2^, the MLV vector failed to infect non-dividing cells (Fig. 7, right). By contrast, the HIV-1 vector efficiently infected both dividing and non-dividing cells (Fig. 7, left).

Collectively, gene disruption of *TRIM5* is a feasible method for transducing bovine cells including non-dividing cells with an HIV-1-based lentiviral vector.

## Discussion

In this study, we utilized multiple approaches to overcome the limited applicability of HIV-1-based lentiviral vector in bovine cells. First, we observed that bovine TRIM5 is the major anti-HIV-1 host factor in MDBK cells. We revealed that the SPRY domain determined the broad anti-lentiviral activity. Second, we found that CA mutants that evade monkey-derived TRIM5 failed to rescue HIV-1 infectivity in MDBK cells. Third, we demonstrated that HIV-1 infection was significantly enhanced by CsA in MDBK cells. Lastly, *TRIM5*-knockout MDBK cells generated in this study displayed significantly higher susceptibility to HIV-1 infection.

Viral vectors are indispensable for broad applications including gene therapy, cancer therapy, and vaccine development^32^. In particular, HIV-1-based lentiviral vectors have a series of advantages compared to other viral vectors. First, because the viral genome integrates into the host genome, the transduced gene can be stably expressed in the host. Second, because HIV-1-based lentiviral vectors can transduce non-dividing cells, they can be applied to broad target cells including neural cells and macrophages. In particular, lentiviral vectors can transduce dendritic cells, leading to the efficient induction of both humoral and cellular immunity to antigens^33^. Therefore, veterinary and agricultural fields might require the application of HIV-1-based lentiviral vectors. Considering the possible application of lentiviral vectors in these fields, stable expression of viral receptors in bovine cells can improve the efficiency of virus isolation from clinical samples, contributing to the better diagnosis and the further understanding of disease pathogenesis.

Consistent with previous studies^6,7^, HIV-1 infection was mainly blocked by bovine TRIM5 in MDBK cells. In addition, bovine TRIM5 suppressed broad lentiviruses including HIV-1, SIVmac, FIV, and EIAV. It is tempting to consider why bovine TRIM5 has such a broad anti-lentiviral spectrum. It had remained unclear which domain of bovine TRIM5 is the determinant for anti-viral activity. In this study, we addressed this point using chimeric molecules between human TRIM5α and bovine TRIM5. We concluded that the SPRY domain of bovine TRIM5 was the major determinant for the species-specific inhibition. It is tempting to elucidate which motif(s) or residue(s) in the SPRY domain determined this specificity in a future study.

Based on previous findings that mutations in CA can change the sensitivity to TRIM5α/TRIMCyp, we tested whether V86M and H87Q mutations rescued HIV-1 infectivity in MDBK cells. Although the H87Q mutation enhanced HIV-1 infectivity in OMK cells and FRhK-4 cells, the mutation failed to rescue HIV-1 infectivity in MDBK cells. This result suggests that the recognition of HIV-1 CA by RM TRIM5α differs from that by bovine TRIM5.

Next, we tested whether modulating CA–CypA binding could rescue HIV-1 infectivity in MDBK cells. We found that the CypA binding-deficient CA mutant RGDA/Q112D + Q4R virus exhibited enhanced infectivity in OMK cells. By contrast, the mutations decreased HIV-1 infectivity in Lenti-X 293T cells. Because Kim et al. demonstrated that CypA protects HIV-1 against human TRIM5α-mediated inhibition^28^, our result might reflect this phenomenon. In MDBK cells, the infectivity of the RGDA/Q112D + Q4R mutant was significantly lower than that of the WT virus, suggesting that these mutations sensitized HIV-1 to bovine TRIM5 or other anti-HIV-1 host factors. We hypothesized that CsA treatment would exert a similar or identical effect as RGDA/Q112D + Q4R mutations. This was true for infection in OMK and Lenti-X 293T cells. However, HIV-1 vector infectivity was threefold higher in CsA-treated MDBK cells than in control cells. This result suggests that CsA relieved the inhibitory effects in MDBK cells. Because CsA targets several Cyps^34,35^, the molecular mechanism of enhancement by CsA should be addressed in a future study. Nevertheless, our finding suggests that CsA treatment is useful when bovine cells need to be transduced by an HIV-1-based lentiviral vector.

Finally, we knocked out *TRIM5* from MDBK cells. The effect of *TRIM5* knockout was obvious, as HIV-1 infectivity was approximately eightfold higher in *TRIM5*-knockout cells. Considering the future use of lentiviral vectors in bovine-derived non-dividing cells, we tested the infectivity of HIV-1 and MLV vectors in *TRIM5*-knockout cells. Although MLV vectors failed to infect non-dividing cells, HIV-1 vectors efficiently infected both dividing and non-dividing cells. This result supports our idea that the application of lentiviral vectors will facilitate gene engineering in bovine cells.

In conclusion, the methods developed in this study provide a promising strategy for gene engineering in bovine cells, contributing to innovations in agriculture and improvements in animal health.

## Materials and methods

### Plasmids

The following plasmids were obtained through the NIH HIV Reagent Program, Division of AIDS, NIAID, NIH: SIV Packaging Construct (SIV3 + , Cat# ARP-13456) and SIV LTR Luciferase mCherry Reporter Vector (Cat# ARP-13455), both of which were provided by Dr. Tom Hope. The pDON-5 Neo DNA plasmid (Cat# 3657) and pLVSIN-CMV Hyg-ZsGreen plasmid (Cat# 6182) were purchased from TaKaRa. The following plasmids were kind gifts from Dr. Kenzo Tokunaga: psPAX2-IN/HiBiT plasmid^36^ and pWPI-Luc2 plasmid^36^. pLionII (Cat# 1730; http://n2t.net/addgene:1730; RRID: Addgene_1730) and pCPRDEnv (Cat# 1732; http://n2t.net/addgene:1732; RRID: Addgene_1732) were gifts from Dr. Garry Nolan. pMD2.G was a gift from Dr. Didier Trono (Cat# 12259; http://n2t.net/addgene:12259; RRID: Addgene_12259). pEIAV-SIN6.1 CGFPW (Cat# 44171; http://n2t.net/addgene:44171; RRID: Addgene_44171)^37^ and pEV53D (Cat# 44168; http://n2t.net/addgene:44168; RRID: Addgene_44168)^38^ were gifts from Dr. John Olsen. pSpCas9(BB)-2A-Puro (PX459) V2.0 was a gift from Dr. Feng Zhang (Addgene plasmid # 62988; http://n2t.net/addgene:62988; RRID: Addgene_62988). To generate a luciferase-encoding FIV vector, a cDNA encoding luciferase 2 (*luc2*) was ligated to the pLionII plasmid. To generate a luciferase-encoding EIAV vector, cDNA encoding *EGFP* in the pEIAV-SIN6.1 CGFPW plasmid was swapped with cDNA encoding *luc2*.

### Cell culture

Lenti-X 293T (TaKaRa, Cat# Z2180N), OMK (American Type Culture Collection (ATCC), Cat# CRL-1556), HeLa (ATCC, Cat# CCL-2), FRhK-4 (ATCC, Cat# CRL-1688), MDBK (Japanese Collection of Research Bioresources Cell Bank (JCRB), Cat# IFO50014), and CRFK cells (JCRB, Cat# JCRB9035) were cultured in Dulbecco’s modified Eagle’s medium (Nacalai Tesque, Cat# 08458–16) supplemented with 10% fetal bovine serum and 1 × penicillin–streptomycin (Nacalai Tesque, Cat# 09367–34).

### Rescue of reporter viruses

To rescue an HIV-1–based lentiviral vector, Lenti-X 293T cells were co-transfected with the psPAX2-IN/HiBiT, pLVSIN-CMV Hyg-ZsGreen or pWPI-Luc2, and pMD2.G plasmids using TransIT-293 Transfection Reagent (TaKaRa, Cat# V2700) in Opti-MEM I Reduced Serum Medium (Thermo Fisher Scientific, Cat# 31985062). To rescue an SIVmac-based lentiviral vector, Lenti-X 293T cells were co-transfected with the pSIV3 + plasmid, SIV LTR Luciferase mCherry Reporter Vector, and pMD2.G plasmid. To rescue an FIV-based lentiviral vector, Lenti-X 293T cells were co-transfected with the pCPRDEnv, pLionII-luc2, and pMD2.G plasmids. To rescue an EIAV-based lentiviral vector, Lenti-X 293T cells were co-transfected with pEV53D, EIAV-SIN6.1-luc2, and pMD2.G plasmids. To rescue an MLV-based retroviral vector, Lenti-X 293T cells were co-transfected with pGP, pDON-5 Neo-luc2, and pMD2.G plasmids. The supernatant was collected and filtered 2 days after transfection.

### Virus infection

CRFK, OMK, FRhK-4, and MDBK cells were plated on a 96-well plate at 1 × 10^4^ cells per well. Lenti-X 293T cells were plated on a 96-well plate at 3 × 10^4^ cells per well. After overnight culture, the cells were infected with the reporter viruses. In case of the infection of non-dividing cells, MDBK cells were plated on a 96-well plate at 2.5 × 10^4^ cells per well and treated with 1 µg/mL Aphidicolin (Aph) (WAKO, Cat# 011–09811) for 24 h prior to infection. As needed, cells were cultured in the presence of 2 µM Cyclosporin A (CsA) (Selleck, Cat# S2286). For infection with HIV-1 CA mutants, the cells were infected with equal amount of viruses (861 HiBiT values per well). The HiBiT value was measured using the Nano Glo HiBiT Lytic Detection System (Promega, Cat# N3040) as described previously^36^.

For ZsGreen-encoding virus, ZsGreen positivity in the infected cells was measured 2 days after infection using an Attune NxT Flow Cytometer (Thermo Fisher Scientific) and a CytKick Autosampler (Thermo Fisher Scientific). For luciferase-encoding virus, the infected cells were lysed 2 days after infection with a Bright-Glo Luciferase Assay System (Promega, Cat# E2620) and the luminescent signal was measured using a GloMax Explorer Multimode Microplate Reader (Promega).

### *TRIM5* depletion

To deplete *TRIM5*, MDBK cells adjusted to 5 × 10^5^ cells per well in a 6-well plate were transfected with siRNA targeting bovine *TRIM5* (5′-AGAAUGAUCUGGUCCAAGA-3′) (synthesized by Sigma-Aldrich) or scrambled negative control siRNA (Thermo Fisher Scientific, Cat# 465372) with TransIT-X2 Dynamic Delivery System (TaKaRa, Cat# V6100) in Opti-MEM. After overnight culture, the cells were re-plated on a new 96-well plate at 1 × 10^4^ cells per well. The cells were cultured again overnight and infected with ZsGreen-encoding lentiviruses. Two days after infection, the ZsGreen-positive rate was measured using an Attune NxT Flow Cytometer.

### Quantification of mRNA levels in depleted cells

MDBK cells transfected with siRNA were plated on a 96-well plate at 1 × 10^4^ cells per well. After overnight culture, mRNA expression was quantified by qRT-PCR using the CellAmp Direct RNA Prep Kit for RT-PCR (Real Time) (TaKaRa, Cat# 3732), One Step TB Green PrimeScript PLUS RT-PCR Kit (Perfect Real Time) (TaKaRa, Cat# RR096A), and primer pairs for bovine *TRIM5* (5′-CCATTTGCAGGGATCAACAATG-3′ and 5′-CTCGAAACACTCTCCTCTGTTC-3′) and *Gapdh* (5′-GCGATACTCACTCTTCTACCTTCGA-3′ and 5′-TCGTACCAGGAAATGAGCTTGAC-3′). qRT-PCR was performed using the QuantStudio 5 Real-Time PCR System (Thermo Fisher Scientific), and the Ct values of *TRIM5* were normalized to the mean values obtained using *Gapdh* as a housekeeping gene (ΔΔCt method).

### Construction of plasmids for expressing TRIM5 molecules

Total RNA was extracted from MDBK and HeLa cells using an RNeasy Mini Kit (QIAGEN, Cat# 74104) and QIAshredder (QIAGEN, Cat# 79656). cDNA was synthesized using SuperScript IV Reverse Transcriptase (Thermo Fischer Scientific, Cat# 18090050) and primers bT5-R (5′-AACGTCGACGGATCCTCAAGCATAATCAGGAACATCATATGGATAACAGCTTGGTGAGCACAGAGTCATG-3′) for total RNA extracted from MDBK cells or hT5-R (5′-AACGTCGACGGATCCTCAAGCATAATCAGGAACATCATATGGATAACAGCTTGGTGAGCACAGAGTCATG-3′) for total RNA extracted from HeLa cells. Note that the underlines in the primer sequences indicate the hemagglutinin (HA) tag (YPYDVPDYA) sequence.

To amplify *TRIM5* cDNA by RT-PCR, we used PrimeSTAR GXL DNA polymerase (TaKaRa, Cat# R050A), bT5-F (5′-TGGGCCCGCGGCCGCGCCACCATGGCTTCAGGAATCCTGATGAACA-3′) and bT5-R for bovine *TRIM5* cDNA or hT5-F (5′-TGGGCCCGCGGCCGCGCCACCATGGCTTCTGGAATCCTGGTTAATG-3′) and hT5-R for human *TRIM5α* cDNA. The PCR protocol consisted of 30 cycles of 98 °C for 10 s, 60 °C for 15 s, and 68 °C for 1 min, followed by 68 °C for 7 min. The amplified fragments were ligated into a pDON-5 Neo vector (TaKaRa, Cat# 3657), which was pre-linearized with NotI-HF (NEB, Cat# R3189L) and BamHI-HF (NEB, Cat# R3136L) using an In-Fusion HD Cloning Kit (TaKaRa, Cat# Z9633N). Then, the plasmid was amplified using NEB 5-alpha F′Iq Competent *E. coli* (High Efficiency) (NEB, Cat# C2992H). The plasmids were extracted with PureYield Plasmid Miniprep System (Promega, Cat# A1222). The sequences of bovine *TRIM5* mRNA (Accession# LC727630) and human *TRIM5α* mRNA (Accession# LC727631) were deposited in the GenBank. To generate a chimeric molecule termed BoHu, we used bT5-F and BoHu-R (5′-CACATCTGTCAGCTCATTAAACACA-3′) to amplify the bovine RBCC domain and BoHu-F (5′-GAGCTGACAGATGTGCGACGCTACTGGGTTGATGTGACAG-3′) and hT5-R to amplify the human SPRY domain. These PCR fragments were mixed and amplified with bT5-F and hT5-R and then cloned into the pDON-5 Neo vector. To generate the chimeric molecule HuBo, we used hT5-F and HuBo-R (5′-GACATCTGTCAGCTCTCTAAACACT-3′) to amplify the human RBCC domain and HuBo-F (5′-GAGCTGACAGATGTCCAACGCTACTGGGTTCAGGTGACCC-3′) and bT5-R to amplify the bovine SPRY domain. These PCR fragments were mixed and amplified with hT5-F and bT5-R and then cloned into the pDON-5 Neo vector. To generate the deletion mutant delSPRY, we used bT5-F and bT5-delSPRY-R (5′-AACGTCGACGGATCCTCAAGCATAATCAGGAACATCATATGGATACACATCTGTCAGCTCATTAAACACA-3′) to amplify the bovine RBCC domain, and the PCR fragment was cloned into the pDON-5 Neo vector. The sequences of all plasmids were verified using a SupreDye v3.1 Cycle Sequencing Kit (M&S TechnoSystems, Cat# 063001) with an Applied Biosystems 3130xl DNA Analyzer (Thermo Fisher Scientific).

### Generation of CRFK cells stably expressing TRIM5 molecules

Lenti-X 293T cells were co-transfected with pDON-5 Neo plasmids encoding each TRIM5 molecule, pGP packaging plasmid (TaKaRa, Cat# 6160), and pMD2.G plasmid with TransIT-LT1 Transfection Reagent (TaKaRa, Cat# V2300) in Opti-MEM. The supernatant was filtrated 2 days after transfection. The collected retroviral vectors were used to infect CRFK cells. The cells were cultured in the presence of 500 µg/mL G-418 (Nacalai Tesque, Cat# 09380–44) for 6 days. Then, single-cell cloning was performed, and the expression of TRIM5 in each clone was evaluated by Western blotting.

### Generation of HIV-1 CA mutants

To generate CA mutants, we performed mutagenesis by overlapping PCR using PrimeSTAR GXL DNA Polymerase. To generate the V86M mutant, we used psPAX2-ClaI-F (5′-GAGAATTAGATCGATGGGAAAAAAT-3′) and V86M-R (5′-CCCTGCATGCATTGGATGCACTCTATCCC-3′) to amplify the 5′ fragment and V86M-F (5′-GTGCATCCAATGCATGCAGGGCCTATTGC-3′) and psPAX2-EcoRV-R (5′-GCACATTGTACTGATATCTAATCCC-3′) to amplify the 3′ fragment. These fragments were mixed and amplified with psPAX2-ClaI-F and psPAX2-EcoRV-R. To generate the H87Q mutant, we used psPAX2-ClaI-F and H87Q-R (5′-AGGCCCTGCTTGCACTGGATGCACTCTAT-3′) to amplify the 5′ fragment and H87Q-F (5′-CATCCAGTGCAAGCAGGGCCTATTGCACC-3′) and psPAX2-EcoRV-R to amplify the 3′ fragment. These fragments were mixed and amplified with psPAX2-ClaI-F and psPAX2-EcoRV-R. The resultant PCR fragments encoding V86M or H87Q were mixed with the psPAX2-IN/HiBiT plasmid, which was predigested with ClaI (NEB, Cat# R0197L) and EcoRV-HF (NEB, Cat# R3195L), and then ligated with the In-Fusion HD Cloning Kit. After the mini prep, the plasmids were verified by sequencing. In addition, the sequence of RGDA/Q112D + Q4R^29^, which is a CA mutant that does not bind to CypA, was used to generate the psPAX2-IN/HiBiT- RGDA/Q112D + Q4R plasmid.

### Screening of sgRNA

To generate *TRIM5*-knockout MDBK cells, we designed five candidate sgRNAs for bovine *TRIM5* on the website of CRISPRdirect (https://crispr.dbcls.jp/) (accessed on 4/4/2022). We used a Guide-it Complete sgRNA Screening System (TaKaRa, Cat# Z2636N) to prepare sgRNAs targeting bovine *TRIM5*. Briefly, PCR was performed using PrimeSTAR Max Premix (2 ×), Guide-it Scaffold Template, and the following oligos: sgRNA #1 (5′-CCTCTAATACGACTCACTATAGGAACAACAAGGAATCCATAATGTTTAAGAGCTATGC-3′), sgRNA #2 (5′-CCTCTAATACGACTCACTATAGGCTTCCCCTCTTGGCCAATTAGTTTAAGAGCTATGC-3′), sgRNA #3 (5′-CCTCTAATACGACTCACTATAGGTCGGAGCCTCTGCACTATATGTTTAAGAGCTATGC-3′), sgRNA #4 (5′-CCTCTAATACGACTCACTATAGGCAGAGGCTCCGAGAGGTTAGTTTAAGAGCTATGC-3′), and sgRNA #5 (5′-CCTCTAATACGACTCACTATAGGTTGGCTTTGTGAGCGATCTCGTTTAAGAGCTATGC-3′). The PCR protocol consisted of 33 cycles of 98 °C for 10 s and 68 °C for 10 s. Next, we mixed sgRNA PCR template, Guide-it In Vitro Transcription Buffer, Guide-it T7 Polymerase Mix, and RNase-free water to transcribe sgRNA from the PCR fragments. The PCR protocol was 37 °C for 4 h. After this reaction, recombinant DNase I (RNase-free) was added to the solution, which was incubated at 37 °C for 15 min. The transcribed sgRNA was purified with a column included in the kit. To test the in vitro cleavage efficiency of each sgRNA, genomic DNA was extracted from MDBK cells using DNeasy Blood & Tissue Kit (QIAGEN, Cat# 69506). Next, we mixed 2 × Terra PCR Direct Buffer, forward primer (5′-AATATTTTGTTCTTGGGTCATCCTT-3′), reverse primer (5′-TGAATGTCTACTTCTGCCTAAGAGC-3′), Terra PCR Direct Polymerase Mix, RNase-free water, and genomic DNA of MDBK to amplify the target DNA. The PCR protocol was 98 °C for 2 min, 35 cycles of 98 °C for 10 s and 60 °C for 15 s, and 68 °C for 1 min. Then, the band was visualized by electrophoresis and purified using a QIAquick Gel Extraction Kit (QIAGEN, Cat# 28706). Transcribed sgRNA and Guide-it Recombinant Cas9 Nuclease were mixed and incubated at 37 °C for 5 min. Next, the target DNA, 15 × Cas9 Reaction Buffer, 15 × BSA, and RNase-free water were mixed and incubated at 37 °C for 1 h, followed by 80 °C for 5 min. The reaction product was electrophoresed on a 1% agarose gel to visualize the cleaved bands.

### Generation of *TRIM5* knockout cells

Based on the result of the in vitro digestion assay, we generated a pSpCas9 (BB)-2A-Puro (PX459) V2.0 plasmid^39^ encoding sgRNA #4. We mixed these oligos: bT5a-4 (5′-caccgGCAGAGGCTCCGAGAGGTTA-3′), bT5a-4as (5′-aaacTAACCTCTCGGAGCCTCTGCc-3′), and water and heated the mixture at 95 °C for 5 min, followed by incubation at room temperature for 1 h for oligo annealing. The mixture was 250-fold diluted with water and used for ligation with the PX459 V2.0 plasmid, which was predigested with BbsI-HF (NEB, Cat# R3539L). The solution was mixed with DNA Ligation Kit < Mighty Mix > (TaKaRa, Cat# 6023) and used for transformation with NEB 5-alpha F'Iq Competent *E*. *coli*. After the miniprep, the nucleotide sequence of the plasmid was verified using a primer (5′-ACTATCATATGCTTACCGTAAC-3′).

The PX459-TRIM5-sgRNA#4 plasmid was used for transfection on MDBK cells using TransIT-X2 Dynamic Delivery System. MDBK cells were cultured for 2 weeks in the presence of 1 µg/mL puromycin (InvivoGen, Cat# ant-pr-1). The cells were single-cell-cloned using a limiting dilution method. Each clone was characterized by viral infection and sequencing of genomic DNA as described previously.

### Western blotting

The pelleted cells were lysed in 2 × Bolt LDS sample buffer (Thermo Fisher Scientific, Cat# B0008) containing 2% β-mercaptoethanol (Bio-Rad, Cat# 1610710) and incubated at 70 °C for 10 min. Expression level of HA-tagged TRIM5 in CRFK cells was confirmed by Western blotting using an anti-HA Tag (6E2) mouse mAb (HRP Conjugate) (CST, Cat# 2999S, × 5,000). After stripping of the membrane using WB Stripping Solution Strong (Nacalai Tesque, Cat# 05677–65), the membrane was re-probed with an anti-β-Actin–HRP antibody (Sigma-Aldrich, Cat# A3854-200UL, × 20,000) as a loading control. Chemiluminescence was detected using Western BLoT Ultra Sensitive HRP Substrate (TaKaRa, Cat# T7104A) according to the manufacturer’s instructions. Bands were visualized using an iBright FL1500 imaging system (Thermo Fisher Scientific), and the band intensity was quantified using iBright analysis software (Thermo Fisher Scientific).

### Alignment of bovine TRIM5 and human TRIM5α proteins

The protein sequences of bovine TRIM5 (Accession# LC727630) and human TRIM5α (Accession# LC727631) were aligned using the MUSCLE algorithm on MEGA X (MEGA Software). The parameters of alignment were as follows: gap open, − 2.90; gap extend, 0.00; and hydrophobicity multiplier, 1.20.

### Calculation of the identity between bovine TRIM5 and human TRIM5α proteins

The identity between bovine TRIM5 (Accession# LC727630) and human TRIM5α (Accession# LC727631) proteins was calculated on the website of SIM—Alignment Tool for protein sequences (https://web.expasy.org/sim/) (accessed on 7/11/2022). The parameters of calculation were as follows: comparison matrix, BLOSUM62; number of alignments computed, 20, gap open penalty; 12, and gap extension penalty, 4.

### Statistical analysis

Differences in infectivity between two different conditions (e.g., between DMSO and Aphidicolin) were evaluated by an unpaired, two-tailed Student’s *t*-test. Differences in infectivity between WT, V86M, and H87Q were evaluated by one-way ANOVA, followed by the Tukey test. *p* ≤ 0.05 were considered statistically significant. These tests were performed using Prism 9 software v9.1.1 (GraphPad Software).

## Supplementary Information

Supplementary Information.

## Data Availability

The datasets generated and/or analysed during the current study are available in the GenBank (Accession# LC727630 for bovine *TRIM5* mRNA isolated from MDBK cells, and LC727631 for human *TRIM5α* mRNA isolated from HeLa cells, respectively).
