# Supplementary material for: Generation of a bovine cell line for gene engineering using an HIV-1-based lentiviral vector
Source: Sci Rep. 2022 Oct 18;12:16952. doi: 10.1038/s41598-022-20970-6 (PMC9579131; doi:10.1038/s41598-022-20970-6)
Supplement: Supplementary file 1 — Supplementary Information. [file 41598_2022_20970_MOESM1_ESM.docx]

**SUPPLEMENTARY DATA**

**Generation of a bovine cell line for gene engineering using an HIV-1-based lentiviral vector**

Nanami Morizako ^1^, Erika P Butlertanaka ^1^, Yuri L Tanaka ^1^, Honoka Shibata ^1^, Tamaki Okabayashi ^1,2,3^, Hirohisa Mekata ^2^, Akatsuki Saito ^1,2,3^ *

*^1^Department of Veterinary Science, Faculty of Agriculture, University of Miyazaki, Miyazaki, Miyazaki 8892192, Japan*

*^2^Center for Animal Disease Control, University of Miyazaki, Miyazaki, Miyazaki 8892192, Japan*

*^3^Graduate School of Medicine and Veterinary Medicine, University of Miyazaki, Miyazaki, Miyazaki 8891692, Japan*

*Address correspondence to Akatsuki Saito ([sakatsuki@cc.miyazaki-u.ac.jp](mailto:sakatsuki@cc.miyazaki-u.ac.jp))

**
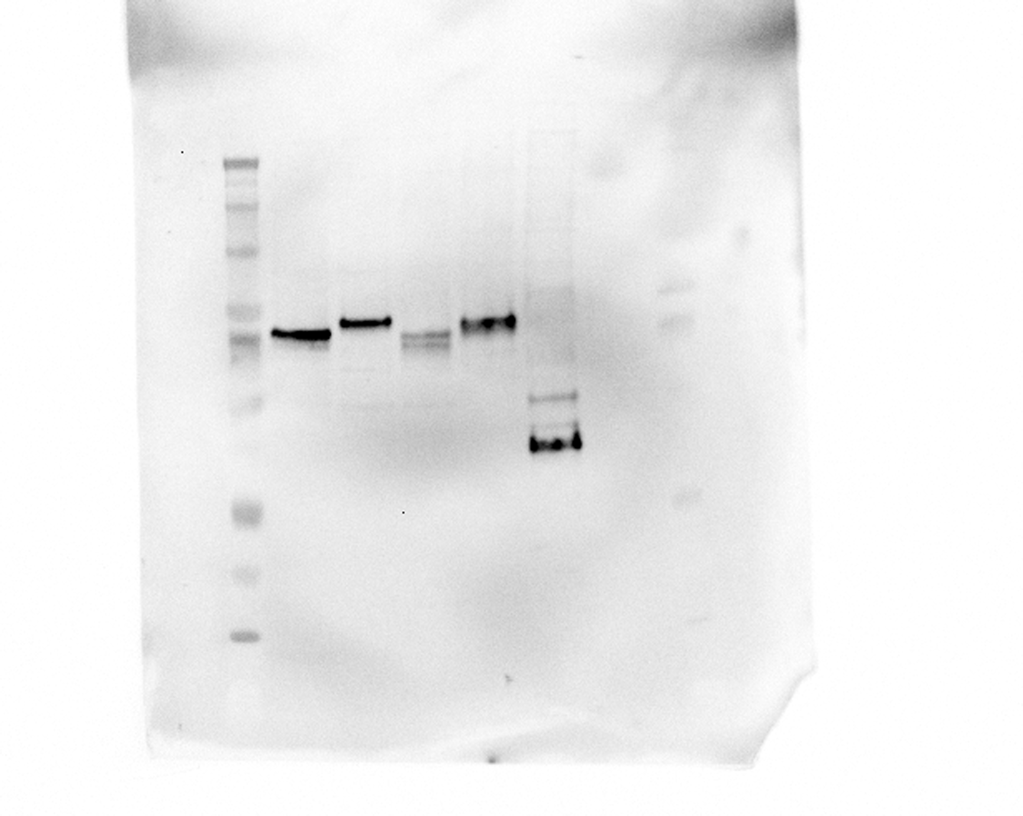
a**

**
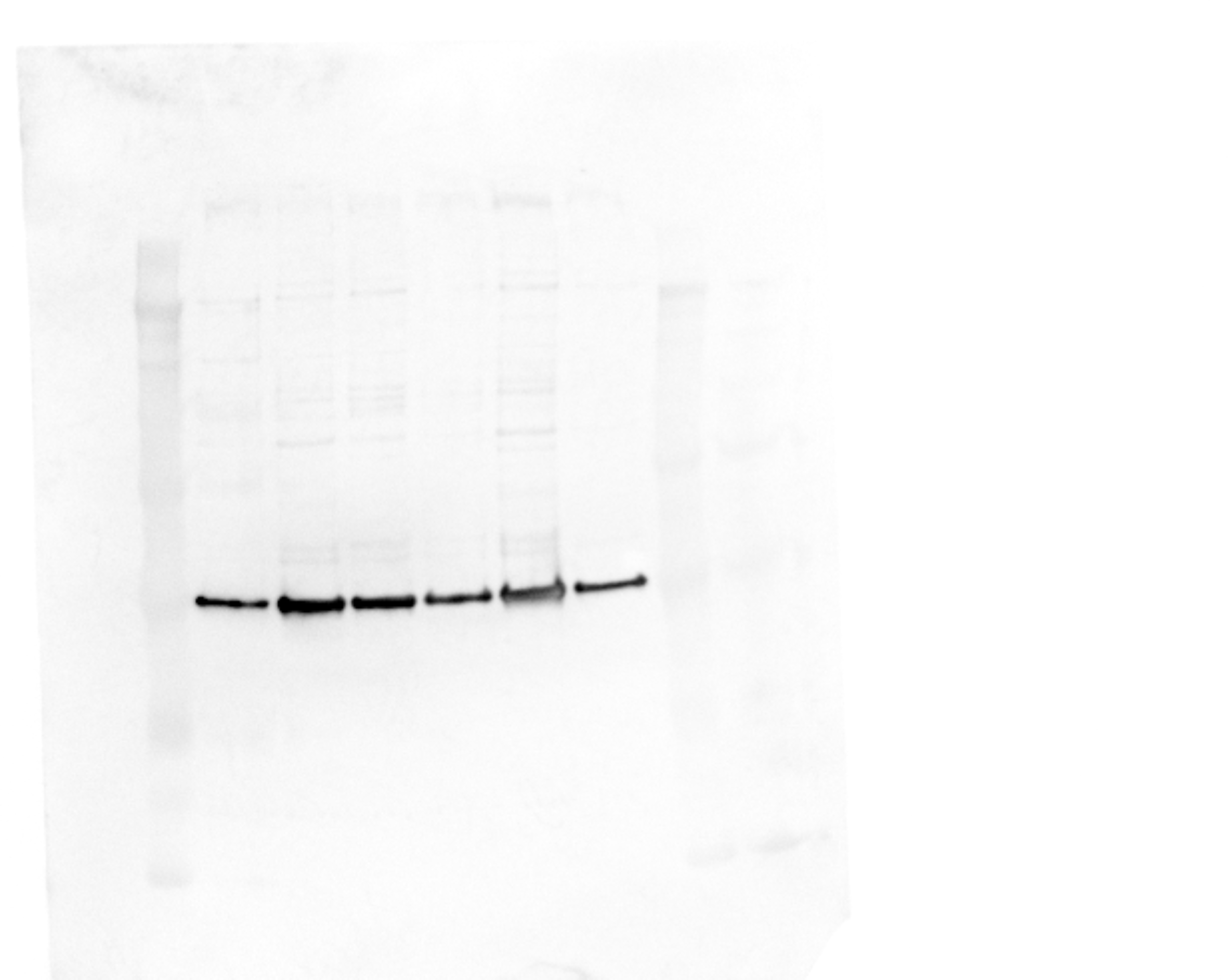
**

**b**

**Supplementary Figure 1.** **Western Blotting of CRFK cells stably expressing TRIM5 molecules.** **(a)** Complete blot for **Figure 2c**. The membrane was probed with an anti-HA antibody. **(b)** The same membrane used in (a) was stripped, and then re-probed with an anti-β-Actin–HRP antibody.
